# *Wohlfahrtiimonas chitiniclastica*: a potential disruptor of wound healing and glucose metabolism in diabetic foot ulcers

**DOI:** 10.3389/fmicb.2026.1837891

**Published:** 2026-07-09

**Authors:** Theresa Kurze, Muhammed Afthab Tharakathel Abdul Majeed, Percy Schröttner, Hani Harb

**Affiliations:** 1 Institute for Medical Microbiology and Virology, Faculty of Medicine and University Hospital Carl Gustav Carus, Technische Universität Dresden, Dresden, Germany; 2 Institute for Clinical Chemistry and Laboratory Medicine, Faculty of Medicine and University Hospital Carl Gustav Carus, Technische Universität Dresden, Dresden, Germany

**Keywords:** diabetes, diabetic ulcers, immune reaction, *Wohlfahrtiimonas chitiniclastica*, wound healing

## Abstract

**Introduction:**

The growing global awareness of bacterial pathogens is highlighting previously underestimated organisms and their contribution to established infections. *Wohlfahrtiimonas chitiniclastica* has been lately identified in chronic, non-healing diabetic foot ulcers (DFUs), highlighting its emerging role in these infections.

**Methods:**

To explore host-pathogen interactions, an epithelial cell line A549 and monocyte cell line THP-1 cells were infected with *W. chitiniclastica.* Gene expression profiling was conducted. To mimic diabetic conditions, infections were performed under varying glucose levels, followed by PCR for cytokine analysis. To assess potential effects on wound healing, a scratch assay was performed on epithelial cells infected with *W. chitiniclastica* for 24 h, using a Keyence BZ-X800 microscope for analysis.

**Results:**

While no significant direct effect on epithelial cells was detected, the lack of a beneficial cellular response may still promote delayed wound healing and thereby contribute to disease progression. However, it elicited a pronounced activation of THP-1 monocytes, characterized by elevated cytokine production. Notably, cytokine expression in THP-1 cells was attenuated under high-glucose conditions. Surprisingly, the scratch assay revealed a significant delay in cell line closure in the presence of all *W. chitiniclastica* isolates or their supernatants. Additionally, all strains demonstrated robust growth in different glucose concentration environments, with no significant differences observed.

**Conclusion:**

These findings suggest that *W. chitiniclastica* modulates immune responses in a glucose-dependent manner, potentially enabling immune evasion in the inflammatory environment of DFUs. *W. chitiniclastica* isolates significantly delayed wound closure, indicating a potential role in impaired chronic wound healing. Furthermore, *W. chitiniclastica*’s ability to grow in hyperglycaemic conditions without classical fermentation indicates oxidative glucose metabolism and a survival advantage.

## 1 Introduction

*Wohlfahrtiimonas chitiniclastica* is a Gram-negative, strictly aerobic and non-motile Gammaproteobacterium that was first isolated from the larvae of the parasitic fly *Wohlfahrtia magnifica* (Toth et al., 2008). *W. magnifica*, originally described in 1962, is an obligate parasitic fly that deposits its eggs and larvae into wounds of mammals and humans (Robbins and Khachemoune, 2010). This infestation, known as myiasis, is defined as the invasion of living humans or vertebrates by dipteran larvae that feed on living or dead tissue, body fluids, or ingested food of the host (Robbins and Khachemoune, 2010). *W. chitiniclastica* is characterized by catalase and oxidase positivity, while biochemical testing for urease, indole, and H₂S is negative (Toth et al., 2008). Its optimal growth temperature ranges between 28 and 36 °C, which facilitates bacterial proliferation in traumatic skin lesions or mucosal surfaces contaminated by fly larvae (Toth et al., 2008; Robbins and Khachemoune, 2010; Rebaudet et al., 2009; Ruiz-Martinez et al., 1989; Thaiwong et al., 2014). The strong chitinase activity of the bacterium appears to support larval metamorphosis and reflects a close symbiotic association with the host fly (Tóth et al., 2001).

Furthermore, antimicrobial susceptibility testing showed that the bacterium is susceptible to *β*-lactam antibiotics such as ampicillin, amoxicillin, piperacillin, carbapenems, cephalosporins, and monobactams, as well as to fluoroquinolones (Kopf et al., 2021). In contrast, resistance has been reported to aminoglycosides such as amikacin, tobramycin, and gentamicin, and a natural resistance to fosfomycin is suspected (Matos et al., 2019; Schröttner et al., 2017). Effective therapeutic options reported in clinical cases include trimethoprim/sulfamethoxazole, levofloxacin, and cephalosporins such as cefuroxime (Rebaudet et al., 2009; Bueide et al., 2021; Chavez et al., 2017; Connelly et al., 2019; Katanami et al., 2018; Snyder et al., 2020; Campisi et al., 2015; Suryalatha et al., 2015).

According to the case reports published to date, infections are most commonly associated with chronic or necrotic wounds, particularly in patients with diabetes mellitus or underlying vascular pathologies (Kopf et al., 2024). Poor hygienic conditions represented another major risk factor, particularly among homeless individuals, where they were frequently observed (Rebaudet et al., 2009; Schröttner et al., 2017; Almuzara et al., 2011; Fenwick et al., 2019; Harfouch et al., 2021; Leeolou et al., 2021; Nogi et al., 2016). *W. chitiniclastica* has been identified in infections such as osteomyelitis, bacteremia, and sepsis, but can also occur in wounds without larval infestation, suggesting that direct wound contamination is possible (Rebaudet et al., 2009; Almuzara et al., 2011; Kõljalg et al., 2015; Choi et al., 2022). Advances in diagnostic methods, such as MALDI-TOF MS and 16S rRNA sequencing, enable accurate identification and form the basis for reliable epidemiological analyses (Robbins and Khachemoune, 2010; Schröttner et al., 2017). Given its apparent worldwide distribution, further research is required to better define its pathogenicity, epidemiology, and clinical relevance.

## 2 Materials and methods

### 2.1 Bacterial strains

The *W. chitiniclastica* strains used in this study were provided by the Leibniz Institute DSMZ – German Collection of Microorganisms and Cell Cultures GmbH (Braunschweig, Germany) as lyophilized samples. Rehydration was performed according to instructions provided by the DSMZ DSMZ instructions, and the pellets were resuspended in 500 μL Müller-Hinton Broth and streaked onto Columbia Blood Agar with 5% sheep blood (bioMérieux, Nürtingen, Germany). Plates were then incubated at 37 °C for an overnight culture of approximately 18 h 24 h. Each strain was stored in three cryotubes (Pro-Lab Diagnostics Inc., Ontario, Canada) at −80 °C and subcultured weekly to maintain culture viability and purity. For comparative purposes, the type strain DSM 18708^T^ described by Toth et al. (2008), originally isolated from the third-stage larva of *Wohlfahrtia magnifica* in Mezöfalva, Hungary, was included (Toth et al., 2008). The clinical isolates DSM 105838, DSM 106597, and DSM 100374 (Toth et al., 2008) were obtained from wound swabs of patients with diabetic foot syndrome at the University Hospital Carl Gustav Carus, Dresden, Germany (Kopf et al., 2021).^1^ Risk factors such as smoking, alcohol abuse, and poor personal hygiene were noted in these patients, consistent with previous observations (Kopf et al., 2021).

Additionally, reference strains of *E. coli* ATCC 25922, *Staphylococcus aureus* ATCC 25923 and *Pseudomonas aeruginosa* ATCC 27853 were obtained from the American Type Culture Collection (ATCC, Manassas, Virginia, USA) and cultured under identical conditions.

Frozen colonies were grown on Colombia blood agar plates (bioMérieux, Nürtingen, Germany) for 24 h at 37 °C. A single colony from each isolate was picked and transferred to a new Colombia blood agar plate. Prior to infection, bacteria were regrown at 37° C in Luria-Bertani Broth (Becton, Dickinson and Company, Heidelberg, Germany) under shaking conditions (100 rpm) in a bacteria 1.5 mL shaker (Eppendorf SE, Hamburg, Germany) for 6 h until reaching logarithmic phase. This standardization and characterization of the logarithmic phase via growth curve measurements allowed for comparability between strains by defining a uniform reference point and aligning analyses to a consistent virulence-associated stage.

All strains were previously isolated in the course of routine diagnostics at the Institute of Medical Microbiology and Virology at the Carl Gustav Carus University Hospital in Dresden. The clinical metadata were published in previous studies by our group. The use of patient data was approved by the Ethics Committee at the Technical University Dresden (EK 61022019).

### 2.2 Cell line and culture conditions

The human alveolar epithelial cell line A549 (ATCC CCL-185; Merck KGaA, Darmstadt, Germany) and the human monocytic cell line THP-1 were selected as an established model for infection experiments (ATCC, Manassas, Virginia, USA). Cells were routinely maintained in T-75 cell culture flasks (Merck KGaA, Darmstadt, Germany) containing 20 mL of complete culture medium, consisting of RPMI-1640 (Roswell Park Institute Medium, Merck KGaA, Darmstadt, Germany) medium supplemented with 10% fetal calf serum (FCS, Sigma-AldrichCo., St. Louis, Missouri, USA) and a penicillin–streptomycin antibiotic mixture (P/S: final concentrations: 50 μg/mL penicillin, 50 μg/mL streptomycin; Gibco ™ by Thermo Fisher Scientific Inc., Darmstadt, Germany). Cultures were incubated at 37 °C under 5% CO₂ in a humidified atmosphere. The incubator (Thermo Fisher Scientific Inc., Darmstadt, Germany) humidity of approximately 95% was maintained by a water tray system. Prior to infection experiments, A549 cells were seeded into 48-well plates (Corning Inc., Corning, NY 14831, USA) at a density of 1 × 10^6^ cells per ml in complete medium containing 10% FCS and P/S. A549 cells were used as a surrogate for epithelial cells as main site of infection and their ability to express alarmins.

As for the THP-1 cells, we used them as a monocyte cell line and were not differentiated into mactophages.

### 2.3 Infection of cells with *W. chitiniclastica*

To investigate the cellular response to *W. chitiniclastica*, A549 and THP-1 cells were infected with bacteria in their logarithmic growth phase as previously defined as 6 h using overnight cultures in complete culture media. An MOI (multiplicity of infection; Gálvez et al., 1997) of 50 was used, corresponding to 50 bacteria per target cell, ensuring a robust interaction between bacteria and host cells. An MOI of 50 was selected to ensure sufficient bacteria–host cell interaction, as higher bacterial loads increase the likelihood of bacterial contact with host cells. Furthermore, previous studies (Ding et al., 2023) demonstrated that certain host signaling pathways require higher MOIs for activation. The defined MOI also provides a standardized and reproducible infection condition across experiments. Bacterial concentrations were estimated using Light Scattering Units (LSU; Orion Diagnostica, Finland) and adjusted according to a previously established LSU-to-cell count calibration. A bacterial inoculum equivalent to 5 × 10^7^ *W. chitiniclastica* cells per well was used, resulting in an MOI of 50:1. Cells were incubated under standard conditions for 6 and 24 h, after which supernatants and cell lysates were collected for downstream analyses, including RNA isolation and gene expression studies. *E.coli* (ATCC 25922) was used as the positive control strain with an MOI of 50 The *E. coli* strain was selected due to its prior application in well-established inflammatory response models, allowing comparison with previously published data (Cuyàs et al., 2025). The negative control was served without any bacteria. The cultures were incubated at 37 °C and 5% CO_2_ with high humidity. All experiments were conducted in triplicates in at least two independent experiments.

### 2.4 RNA-isolation, cDNA-synthesis, and qPCR

Both A549 and THP-1 cell lines were lysed immediately after infection in TRIzol (Sigma Aldrich, St. Louis, Missouri, USA) and homogenized to achieve complete cell disruption. RNA was extracted through phase separation with chloroform (Pan Reac AppliChem ITW Reagents, Darmstadt, Germany) precipitated with isopropanol (VWR® BP HChemicals, Radnor, Pennsylvania, USA), washed with 75% ethanol (Thermo Fisher Scientific Inc., Darmstadt, Germany), and resuspended in RNase-free water. Purity and concentration were confirmed via spectrophotometry using the NanoPhotometer® NP80 (IMPLEN GmbH, München, Germany) to assess the absorbance ratios (A260/A280 ≥ 1.8, A260/A230 > 2.0; Wilfinger et al., 1997).

cDNA was synthesized from 1,000 ng total RNA using the RevertAid First Strand cDNA Synthesis Kit (Thermo Fisher Scientific, Darmstadt, Germany). The resulting cDNA was diluted to 10 ng/μL for quantitative real-time PCR (qPCR). Gene expression was normalized to glyceraldehyde-3-phosphate dehydrogenase (GAPDH; Liang et al., 2007; Pinto et al., 2020). Quantitative Real-Time PCR was performed in triplicate using Real-Power SYBR® green PCR Mastermix (applied biosystems by Thermo Fisher Scientific Inc., Darmstadt, Germany) on CFX96 Touch Real-Time PCR Detection System (Bio-Rad® Laboratories Inc., Hercules, CA, USA). Threshold cycle (CT) values were obtained to establish the relative RNA levels of the tested genes, using GAPDH gene as a housekeeping gene and then calculated with the 2 − DDCt method.

### 2.5 Infection on THP-1 cells with high glucose environment

For subsequent experiments with exposure to different glucose concentrations, two media formulations were prepared: RPMI-1640 medium with 25 mM glucose and RPMI 1640 medium with 25 mM mannitol as an osmotic control. For each condition, 250,000 THP-1 cells were subcultured in 1 mL of the respective medium per well in a 24-well plate. Triplicates were prepared for each bacterial strain in both the glucose and mannitol media. After 3 days of treatment with glucose- or mannitol-containing medium, the cells were infected with bacteria at a multiplicity of infection (MOI) of 50. At this time, cell density was reassessed using a Neubauer-Improved counting chamber (Paul Marienfeld GmbH& Co. KG, Lauda Königshofen, Germany) to ensure accurate MOI adjustment. Each experiment was performed at least twice in triplicate. Following infection, gene expression was analyzed in the same manner as the previously described in RNA isolation, cDNA synthesis and qPCR.

### 2.6 Glucose growth curves

Overnight cultures of the *W. chitiniclastica* strains were prepared on Columbia Blood Agar plates. The following day, colonies were adjusted to 0.5 McFarland units and dissolved in Müller Hinton broth (Becton, Dickinson and Company, USA). A glucose stock solution was prepared by dissolving glucose in double-distilled water (ddH2O). A two-fold serial dilution was performed to generate concentrations ranging from 1,024 μg/mL to 0.5 μg/mL. For each dilution step, 500 μL of the previous solution was mixed with 500 μL of sterile water, halving the concentration at each step. For results only 1,024, 512, 64, 8, 4, and 0.5 μg/mL are shown for the *W. chitiniclastica* strains.

For measurement 50 μL of the standardized bacterial suspension and 50 μL of the respective glucose solution were transferred into the wells of a 96-well microtiter plate (Corning Inc., Corning, NY 14831, USA) The final glucose concentrations in the wells were calculated after mixing equal volumes of bacterial suspension and glucose solution, resulting in a two-fold dilution of the initial glucose concentrations. The plate was placed in a microplate reader, and optical density at 600 nm (OD600, Synergy H1, BioTek Intruments Inc., Vermont, USA) in intervals of 30 min at constant growth conditions at 37 °C und 5% CO_2_ under continous double orbital shaking was measured continuously for at least 17 h to generate growth curves. The growth curves were compared with a glucose-free control („growth control“) condition to assess the influence of glucose availability on bacterial growth. Additionally, growth curves of *Escherichia coli* and *Staphylococcus aureus* strains were included for comparative analysis. All tested concentrations were performed in duplicate, and the glucose growth curve experiments were independently repeated three times.

### 2.7 Scratch assay

For the cell migration assay, A549 cells were subcultured and seeded in 24-well plates (Corning Inc., Corning, NY 14831, USA) at an optimized density of 2.5 × 10^6^ cells per well, using only the inner wells for imaging. Cells were incubated for 24 h under standard conditions to achieve full confluence and stable adhesion. To create a defined wound, the medium was removed, cells were washed with 200 μL phosphate-buffered saline PBS (Merck KGaA, Darmstadt, Germany), and a scratch was introduced along the 6–12 o’clock axis using a 200 μL pipette tip. The cells were washed again with PBS and checked under the microscope to confirm straight edges. Subsequently, 450 μL of HEPES-buffered medium (10 μg/mL HEPES, antibiotic-free, Gibco by Thermo Fisher Scientific Inc., Darmstadt, Germany) was added to each well.

*W. chitiniclastica* strains were prepared by incubating for 5 h to reach the log-phase growth. After centrifugation at 3,500 × g for 5 min at 4 °C (GTR2, Fisher Scientific International Inc., Darmstadt, Germany), 100 μL of the bacterial supernatant was added to each well containing the scratched cells and HEPES medium. Plates were then incubated in BZ-X800 microscope incubation chamber (Keyence, Frankfurt am Main, Germany) at 37 °C with humidity control for 24 h. The supernatants were analyzed for bacterial viability after 72 h to confirm that no bacterial growth occurred under the experimental conditions. Supernatants were plated on bacterial growth medium and incubated to assess potential bacterial regrowth. No sterile filtration of the supernatants was performed in this study. Such a step could have been implemented to remove residual viable bacteria and ensure that subsequent analyses specifically reflected the effects of bacteria-derived soluble factors rather than the presence or growth of viable bacteria. Imaging was performed to obtain focused images, and images were captured hourly from preselected points.

Wound closure was analyzed using the BZ-X Analyzer software (Keyence, Frankfurt am Main, Germany). For each image, 20 distances were measured at regular intervals, averaged, and the percent changes relative to the initial measurement were calculated. These measurements were evaluated over 4-h intervals and summed to determine the Area Under the Curve (AUC), providing a quantitative assessment of cell migration (Liang et al., 2007; Pinto et al., 2020).

### 2.8 Statistical analysis

All the experiments were performed in triplicates in at least two independent experiments and the statistical significance was determined by using Student’s twotailed *t*-test and two-way ANOVA at *p* < 0.05 using the GraphPad Prism 6.0 (GraphPad Software, CA, USA). Assumptions for parametric testing were evaluated prior to applying the statistical analyses.

## 3 Results

### 3.1 Effects of *Wohlfahrtiimonas chitiniclastica* on the expression of Alarmins

First, the results of the interaction between the bacteria and epithelial cells are presented, as epithelial cells represent the primary barrier and protective layer of the body. These cytokines were investigated because they provide insights into inflammatory responses and may indicate a potential delay in wound healing in diabetic foot ulcers (DFUs).

**Figure 1:**
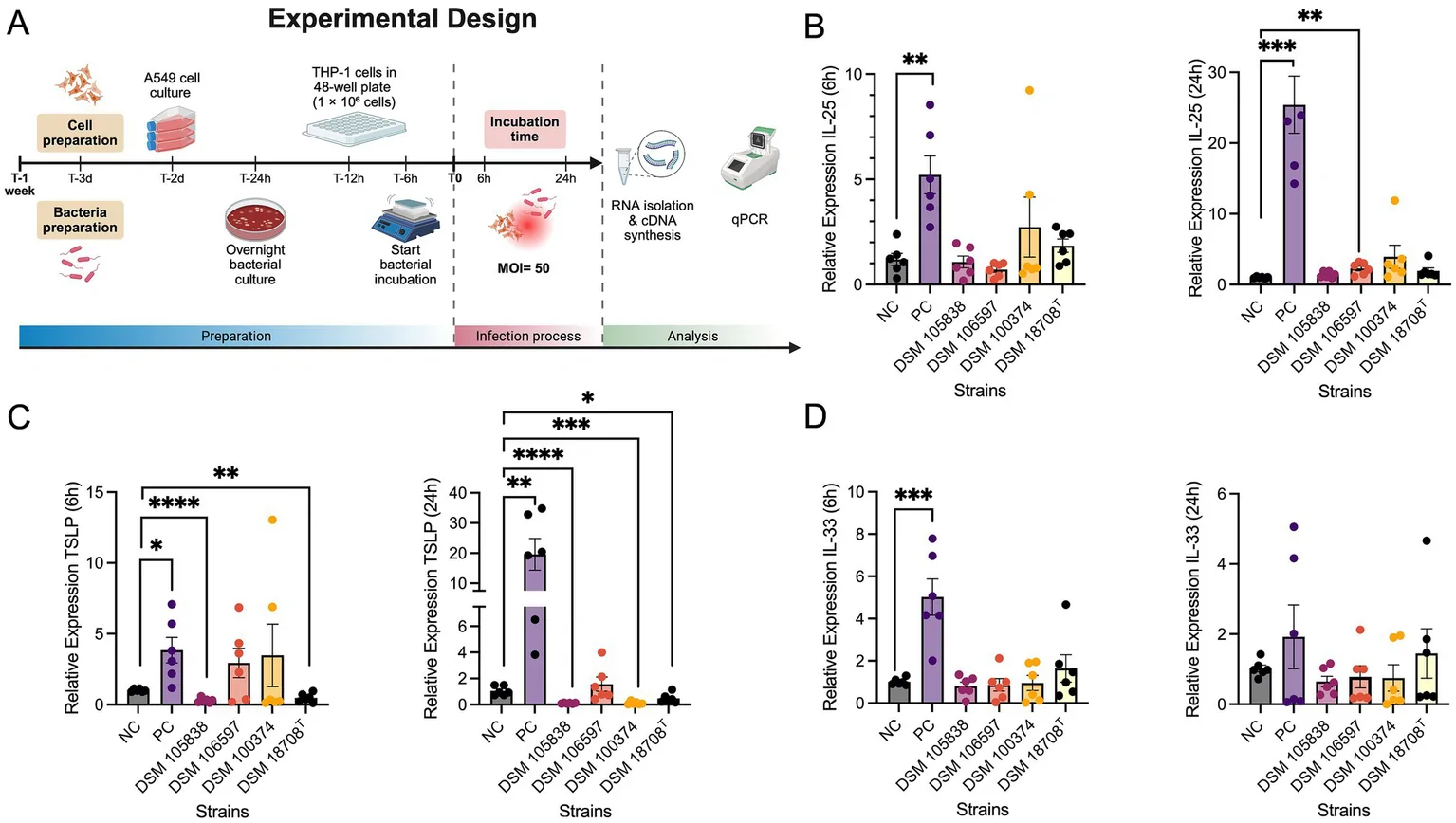
*W. chitiniclastica* induces alarmin gene expression in A549 cells. Experimental design **(A)**. Relative mRNA expression of TSLP **(B)**, IL-25 **(C)**, and IL-33 **(D)** in A549 cells infected with *W. chitiniclastica* (MOI 50) for 6 h and 24 h. Untreated cells served as negative control (NC); *E*. *coli*–infected cells as positive control (PC). Gene expression was analyzed by qPCR using the ΔΔCT method with GAPDH as reference. Data are mean ± SEM (*n* = 6 Biological replicates). Statistical tests: one-way ANOVA with Tukey’s *post-hoc* analysis. Data representative of two independent experiments. *p < 0.05, **p < 0.01, ***p < 0.001, ****p < 0.0001.

The constitutively produced cytokines IL-25, IL-33, and thymic stromal lymphopoietin (TSLP) were analyzed following exposure to *W. chitiniclastica* using quantitative PCR (qPCR; Figure 1A). Uninfected cells served as the negative control, while A549 cells infected with *E. coli* ATCC 25922 were used as the positive control (PC). All experiments were performed in triplicates in at least two independent experiments and evaluated from two independent experiments. Statistical analysis was conducted relative to the negative control (NC).

After an incubation period of 6 h, a significant downregulation of the TSLP gene was observed following infection with *W. chitiniclastica* strains DSM 105838 and DSM 18708^T^ compared to the negative control (Figure 1B). Furthermore, after 24 h, a significant downregulation of TSLP expression persisted in strains DSM 105838 and DSM 18708^T^, similar to the effect observed at 6 h, and was also evident following infection with *W. chitiniclastica* strain DSM 100374 (Figure 1B).

For IL-25 expression, no significant differences compared to the negative control were observed after 6 h of incubation (Figure 1C). After 24 h, however, a significant upregulation was detected in A549 cells infected with *W. chitiniclastica* strain DSM 106597, whereas no significant changes were observed for the remaining strains (Figure 1C).

IL-33 expression did not show significant changes relative to the negative control at either incubation time. Notably, after 24 h, expression levels tended to be below those of the negative control (Figure 1D).

In summary, the expression analysis revealed heterogeneous results. TSLP expression was significantly downregulated at both 6 and 24 h, whereas IL-25 and IL-33 showed only strain-specific and isolated significant changes.

### 3.2 Effects of *Wohlfahrtiimonas chitiniclastica* on the monocytic cell line THP-1 cells

**Figure 2:**
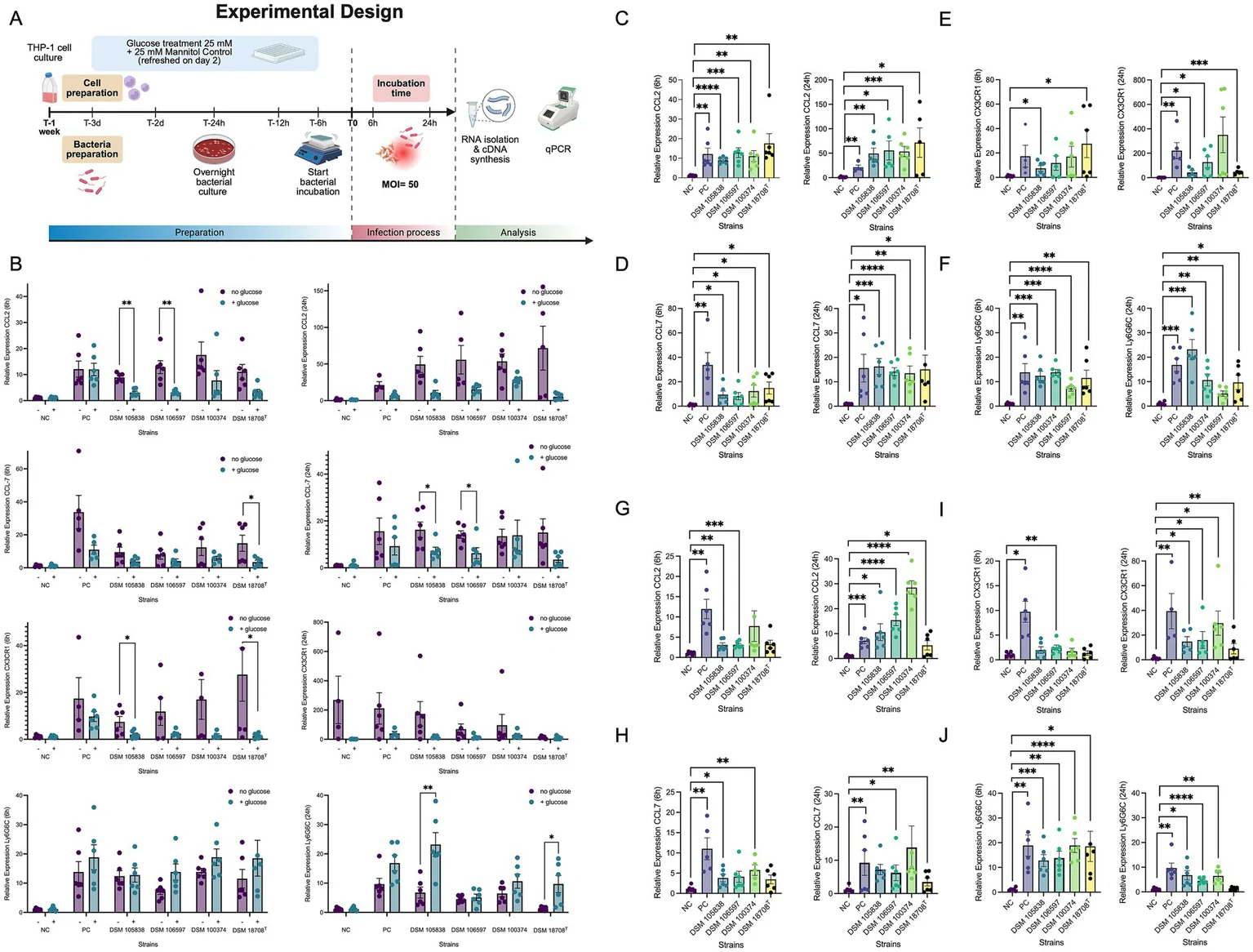
*W. chitiniclastica* induces chemokine and immune marker expression in THP-1 cells under glucose conditions. Experimental design **(A)**. Relative mRNA expression of CCL2 **(C)**, CCL7 **(D)**, CX3CR1 **(E)**, and LY6G6C **(F)** in THP-1 cells infected with *W. chitiniclastica* (MOI 50) for 6 h and 24 h following 3-day pretreatment with 25mMglucose or 25mM mannitol (osmotic control): Direct comparisons between glucose-treated and untreated cells are illustrated **(B)**. Detailed gene expression data for individual genes, shown exclusively for glucose-treated conditions and separated by 6 and 24 h, are provided **(G–J)**. Untreated cells served as negative control (NC); *Escherichia coli*–infected cells as positive control (PC). Gene expression was analyzed by qPCR using the ΔΔCT method with GAPDH as reference. Data are mean ± SEM (*n* = 6 biological Replicates). Statistical tests: one-way ANOVA with Tukey’s *post-hoc* analysis. Data representative of two independent experiments. *p < 0.05, **p < 0.01, ***p < 0.001, ****p < 0.0001.

To better understand inflammatory and infectious mechanisms beyond the skin barrier, the expression of selected chemokines in THP-1 monocytes was analyzed. These chemokines are involved in chemotaxis, inflammatory responses, and may influence DFU healing. The constitutively produced chemokines CCL2, CCL7, CX3CR1, and Ly6G6C were quantified using qPCR following exposure to PAMPs (Figure 2A). Results were statistically evaluated relative to the negative control (NC), while *E. coli* ATCC 25922 served as the positive control (PC).

Both at 6 and 24 h post-infection, CCL2 expression was increased following infection with all *W. chitiniclastica* strains (Figure 2C), with markedly higher expression levels observed after 24 h.

Similarly, CCL7 expression was significantly increased in all infected samples at both incubation times. However, no quantitative difference between the two time points was observed. A more detailed analysis revealed that relative expression levels after 6 h remained below those of the positive control, whereas after 24 h they reached approximately the same level (Figure 2D).

CX3CR1 expression showed heterogeneous results. Significant upregulation was observed following infection with *W. chitiniclastica* strains DSM 105838 and DSM 18708^T^ after 6 h (Figure 2E). After 24 h, significant expression was detected in strains DSM 105838 and DSM 18708^T^, consistent with their upregulation at 6 h, and was also observed following infection with strain DSM 106597. In contrast, infections with strain DSM 100374 showed inconsistent expression patterns at both time points. Overall, a strong tendency toward increased CX3CR1 expression compared to the negative control was observed, particularly after 24 h (Figure 2E).

Ly6G6C expression was significantly upregulated in all infected cells at both incubation times (Figure 2F).

In summary, infection with *W. chitiniclastica* led to a significant overexpression of CCL2, CCL7, and Ly6G6C in THP-1 cells compared to the negative control. CX3CR1 expression also tended to be elevated, although significance was only reached in selected strains.

### 3.3 Effects of glucose treatment on THP-1 cells followed by infection with *Wohlfahrtiimonas chitiniclastica*

In addition to the effect of the regular infection on THP-1 cells the effect on hyperglycemic milieu has to be studied to see whether it influences the pathogenicity of the bacteria toward the cells and mimic the diabetic condition. To investigate the influence of a glucose-rich environment, THP-1 cells were pretreated with a high glucose concentration (25 μM) for 3 days prior to infection (Figure 2A). This approach aimed to mimic altered cellular responses relevant to DFU wound healing. Results were statistically evaluated relative to the negative control (NC), with *E. coli* ATCC 25922 serving as the positive control (PC).

CCL2 expression was significantly increased after 6 h of infection with *W. chitiniclastica* strains DSM 105838 and DSM 106597 (Figure 2G). After 24 h, expression levels exceeded those of the positive control (Figure 2G). However, glucose-pretreated infections showed a significant decrease in CCL2 expression after infection with strains DSM 105838 and DSM 106597 after 6 h compared to infections without glucose (Figure 2B).

CCL7 expression was generally elevated, but only selected strains showed significant decreases: strains DSM 105838 and DSM 100374 at 6 h, and strains DSM 106597 and DSM 18708^T^at 24 h (Figure 2H). Compared to infections without glucose, it was shown a significant decrease on both strain DSM 18708^T^ after 6 h and on strains DSM 105838 and DSM 106597 after 24 h (Figure 2B).

CX3CR1 expression after 6 h remained at the level of the negative control, except for *W. chitiniclastica* strain DSM 106597, which showed a significant increase (Figure 2I). After 24 h, all infected samples exhibited significantly increased expression (Figure 2I), although levels remained significantly below those observed without glucose pretreatment on strain DSM 105838 and DSM 18708^T^ (Figure 2B).

Ly6G6C expression was significantly upregulated at both incubation times in all infections except for strain DSM 18708^T^ at 24 h (Figure 2J). While 6-h expression levels were comparable to infections without glucose, higher expression was observed after the incubation time of 24 h on strains DSM 105838 and DSM 18708^T^(Figure 2B).

In summary, glucose pretreatment resulted in lower expression levels of CCL2, CCL7, and CX3CR1 compared to infections without glucose, whereas Ly6G6C expression was increased especially at 24 h (Figure 2B).

### 3.4 Adaptation of *W. chitiniclastica* to different glucose concentrations

**Figure 3:**
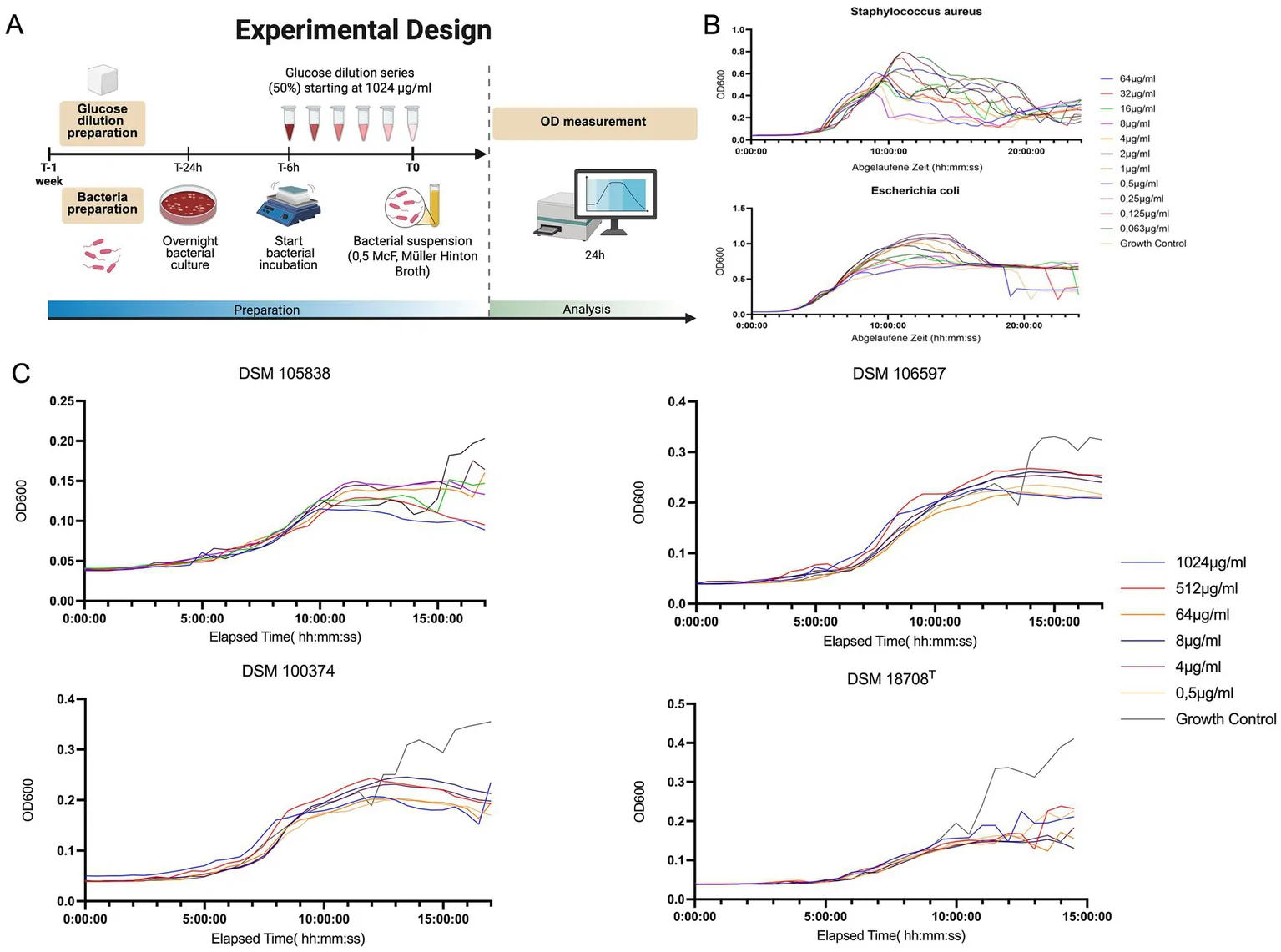
Growth kinetics of *W. chitiniclastica* compared to glucose-utilizing bacteria. Experimental design **(A)**. Growth curves of glucose-utilizing strains *Staphylococcus aureus* and *Escherichia coli* **(B)**. Growth curves of *W. chitiniclastica* strains **(C)**.

In the following experiment we wanted to examine the direct changes and influences of different glucose concentrations rather on the cell infection than on the bacterial growth itself. To create an insight on how glucose is fermented and used throughout the adaptation of the diabetic high glucose environment. To assess glucose adaptation of *W. chitiniclastica*, bacterial strains were exposed to glucose concentrations ranging from glucose-free medium (growth control) to 64 μg/mL in a geometric dilution series. Growth was monitored in Müller–Hinton broth (Becton, Dickinson and Company, USA) and compared to reference strains with known growth characteristics (Figure 3A). *S. aureus* ATCC 25923 and *E. coli* ATCC 25922 served as glucose-fermenting bacteria as controls.

Both *S. aureus* and *E. coli* exhibited exponential growth after a short lag phase, with glucose concentration influencing the timing and magnitude of maximal optical density (OD; Figure 3B).

In contrast, *W. chitiniclastica* strains displayed markedly lower OD increases (maximum ~0.3 units) compared to reference strains. All *W. chitiniclastica* strains showed an approximately exponential increase after ~5 h, followed by a plateau phase. Strains DSM 106597, DSM 100374, and DSM 18708^T^ exhibited similar growth curves independent of glucose concentration, whereas strain DSM 105838 showed a delayed and flatter growth pattern with an earlier plateau at higher glucose concentrations (Figure 3C).

### 3.5 Effects of *W. chitiniclastica* infection on wound closure in A549 cells assessed by scratch assay

An exploratory scratch wound-healing assay was performed to evaluate whether *W. chitiniclastica* influences wound healing (Liang et al., 2007; Pinto et al., 2020). This established *in vitro* method was used to assess cell migration using adherent A549 epithelial cells.

**Figure 4:**
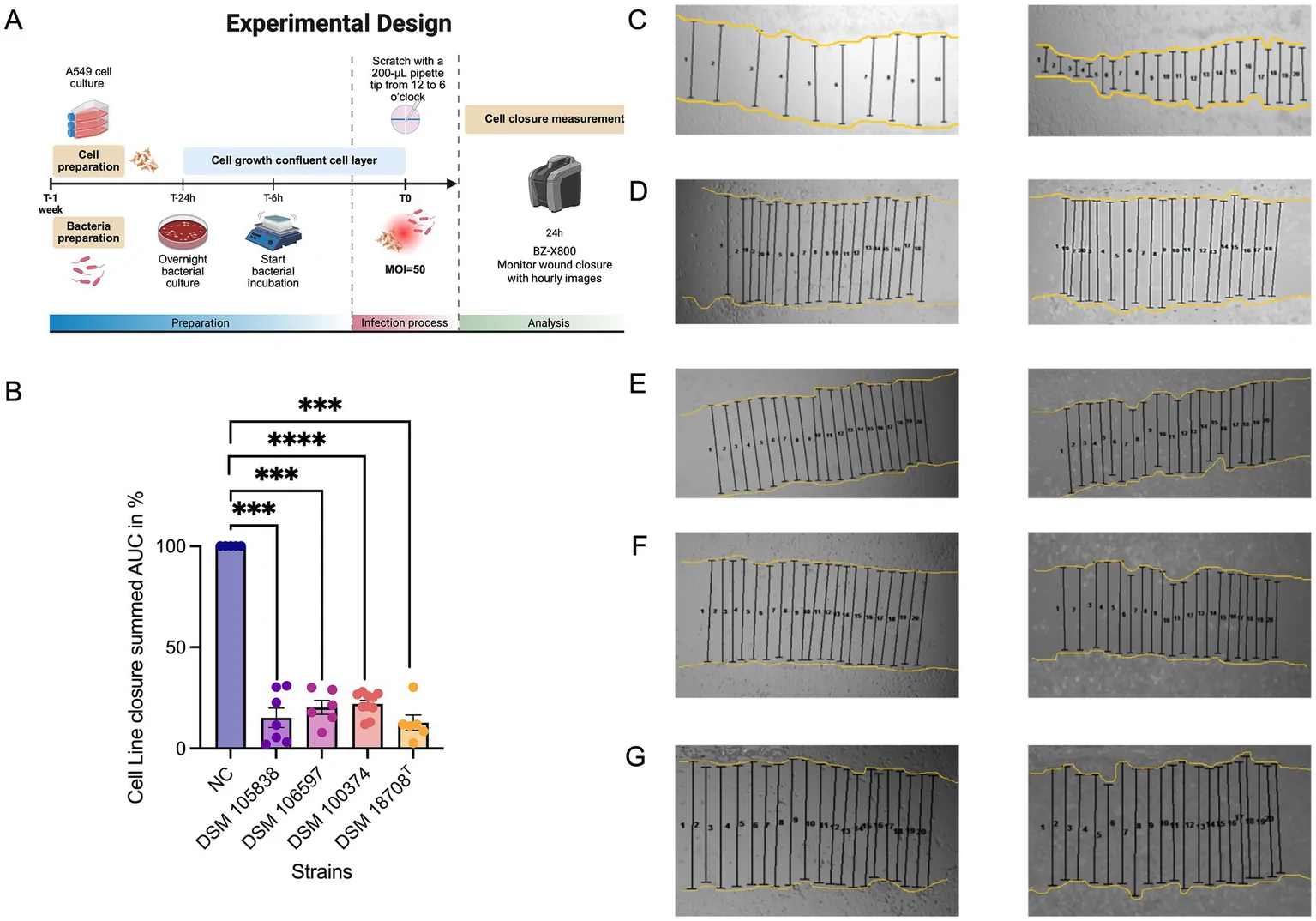
*W. chitiniclastica* modulates epithelial cell migration. Experimental design of the wound healing assay in A549 cells **(A)**. Quantification of wound closure expressed as percentage closure over time and cumulative area under the curve (AUC) **(B)**. Representative images of wound closure at 0 h and 24 h in untreated control cells **(C)** and cells exposed to *W. chitiniclastica* strains DSM 105838 **(D)**, DSM 106597 **(E)**, DSM 100374 **(F)**, and DSM 18708T **(G)**. (*n* = 5 biological Replicates), Statistical tests: one-way ANOVA with Tukey’s *post-hoc* analysis. Data representative of two independent experiments. *p < 0.05, **p < 0.01, ***p < 0.001, ****p < 0.0001.

Cells were monitored over 24 h, and marked regions were photographed at defined intervals. Wound distances were measured and averaged, and cumulative percentage changes in wound area were calculated in four-hour intervals. These values were normalized to the respective negative control and expressed as area under the curve (AUC; Figure 4A).

A significant reduction in wound closure was observed for all *W. chitiniclastica strains.* Strain DSM 100374 showed the lowest, whereas strain DSM 18708^T^ exhibited the highest percentage of wound closure (Figure 4B). Representative images at 0 and 24 h are shown in (Figures 4C–G). Figure 4C depict the uninfected negative control at 0 (left side) and 24 h (right side), respectively.

## 4 Discussion

*W. chitiniclastica* was initially identified in association with myiasis (Toth et al., 2008). Subsequent case reports suggested a broader clinical relevance (Kopf et al., 2024). Moreover, previous studies demonstrated that isolates obtained in Dresden belong to a common subspecies, indicating potential host adaptation (Kopf et al., 2021; Kopf et al., 2022). Earlier work described clinical cases, antibiotic resistance genes, virulence factors, and zoonotic relevance (Kopf et al., 2021; Schröttner et al., 2017; Kopf et al., 2024; Kopf et al., 2022). However, interactions with the immune system and the local wound environment, particularly chronic diabetic foot ulcers, have not been addressed so far.

In this study, we investigated the immune response by *W. chitiniclastica* to various cells to understand their pathomechanism. Initially, we have examined the expression of IL-33, IL-25, and TSLP in A549 epithelial cells following infection with *W. chitiniclastica*, aiming to assess potential effects on wound healing. Infection with *W. chitiniclastica* resulted in a significant downregulation of TSLP, while IL-25 and IL-33 showed no significant induction. The lack of IL-25 induction can impair wound healing markedly (Li et al., 2022). On the other hand, our study has shown that TSLP was significantly downregulated (Figure 1C). This can be linked as well to delayed wound healing through less antiinflammatoric cytokines due to the lack of TSLP (Liu et al., 2019) M2 polarisation, antiinflammatorixc cytokines-delayed wound healing.) Furthermore, He et al. (2017) demonstrated that IL-33 promotes macrophage polarization toward the M2 phenotype, which is critical for tissue repair and regeneration in diabetic mice. Moreover, (Li et al. 2022) reported reduced IL-33 expression in diabetic wound tissue, indicating that impaired IL-33 signaling may contribute to defective wound healing in diabetes (Kopf et al., 2022).

We further investigated the interaction between the bacterium with immune cells. For that we used THP-1 cells as a monocyte model to assess how the expression of chemokines CCL2, CCL7, CX3CR1, and Ly6G6C contributes to proinflammatory pathways that may ultimately influence the healing of diabetic foot ulcers. These chemokines reflect the ability of monocytes to recruit additional monocytes and macrophages to inflammatory sites and initiate key inflammatory processes. After both 6 and 24 h, a highly significant induction of CCL2, CCL7, CX3CR1, and Ly6G6C expression was observed. Previous studies show that CCL2 is closely associated with angiogenesis in diabetic wound healing, as healing wounds exhibit increased endothelial cell numbers and higher CCL2 expression compared to non-healing wounds (Wang et al., 2023). The similar early induction of CCL2 and CCL7, followed by a more pronounced increase after 24 h, suggests coordinated chemoattraction during early inflammation, with enhanced monocyte recruitment at later time points. Alterations in Ly6C^high^ and monocyte populations have been reported, including an accumulation of classical monocytes and delayed differentiation into Ly6C^low^ (Kimball et al., 2018) non-classical subsets (Geissmann et al., 2003; Shi and Pamer, 2011; Yang et al., 2014). Building on its role in monocytic subset differentiation, CX3CR1 signaling-particularly through its ligand CXCL8 (IL-8)—has been linked to non-healing diabetic wounds (Kimball et al., 2018; Geissmann et al., 2003). Elevated IL-8 production is driven by fibroblast phenotype switching induced by proinflammatory cytokines resulting in enhanced macrophage activation and persistent inflammation (Theocharidis et al., 2020; Rai et al., 2022). Furthermore, we observed a significant upregulation of CCL2 and CCL7, indicating a proinflammatory response. Markedly, CX3CR1 expression was strongly elevated after 24 h, whereas Ly6G6C remained consistently high across conditions with minimal temporal variation, except in cells exposed to *W. chitiniclastica* strain DSM 105838. This expression pattern suggests an early predominance of classical inflammatory monocytes with delayed phenotypic switching, in agreement with previous reports (Kimball et al., 2018).

Markedly, all measured inflammatory parameters were significantly increased in THP-1 cells infected with *W. chitiniclastica* under hyperglycemic conditions compared to uninfected controls, similar to infections without glucose supplementation. Furthermore, comparative analysis revealed partially lower expression of CX3CR1, CCL2, and CCL7, whereas Ly6G6C expression was significantly elevated after 24 h. Notably, Ly6G6C expression at 6 h remained high, indicating early dominance of classical inflammatory monocytes. These results suggest that, contrary to the initial hypothesis, CCL2 and CCL7 may be relatively downregulated in glucose-rich environments, indicating that hyperglycemia attenuates their expression despite the expected proinflammatory effect. In this context, previous studies support these findings: CCL2 expression is reduced in diabetic wounds, delaying macrophage response and healing, suggesting hyperglycemia may attenuate CCL2 expression (Wood et al., 2014). Notably, on day 1 post-injury, the expression of CCL2 and other key chemokines was significantly diminished in the wounds of diabetic mice compared to those of normal mice. These results indicate that diabetic wounds are characterized by an impaired chemokine response during the early, acute phase of healing (Roy et al., 2022). Furthermore, the sustained Ly6G6C expression observed here supports the role of classical monocytes with impaired differentiation toward non-classical subsets, corresponding to the murine Ly6C^low^ population (Kimball et al., 2018; Yang et al., 2014). This shift toward an inflammatory monocyte phenotype may contribute to a prolonged inflammatory phase and delayed wound healing, particularly relevant in diabetic foot ulcers.

To investigate the extent to which varying glucose concentrations influence the growth of *W. chitiniclastica* and to derive potential mechanisms by which hyperglycemic wound environments may enhance its pathogenicity or infectivity, 24-h growth curves were recorded and analyzed. Two glucose-fermenting bacterial strains (*S. aureus* and *E. coli* ATCC 25922), and four *W. chitiniclastica* strains were included. Furthermore, all *W. chitiniclastica* strains showed sustained growth under varying glucose concentrations, suggesting their potential to persist in the hyperglycemic environment of diabetic foot ulcers. A fundamental question pertains to the identification of factors that impede bacterial proliferation at elevated glucose levels, and the underlying mechanisms responsible for the unaltered growth curves of *W. chitiniclastica* across the tested range. Observed in studies, bacteria can survive and tolerate in high-glucose environments and are thus found in diabetic foot ulcers, with *S. aureus* most frequently detected in patients with blood glucose between 151 and 200 mg/dL and least frequent at 121–150 mg/dL, while *Klebsiella pneumoniae* showed a similar pattern; overall, bacterial colonization was significantly higher in patients with fasting blood glucose above 150 mg/dL compared to those below 150 mg/dL (Benjamin et al., 2025) Taken together, *W. chitiniclastica* exhibits tolerance to varying hyperglykemic glucose levels, suggesting that this trait may facilitate its persistence in the hyperglycemic environment of diabetic foot ulcers. Whether this tolerance confers a broader survival advantage, and the degree to which the bacterium relies on glucose metabolism, remains to be determined.

Notably, all *W. chitiniclastica* strains used in this study, with the exception of DSM 18708ᵀ, were directly obtained from diabetic foot ulcers. Delayed wound healing has increasingly been attributed in the literature to alterations in inter- and intracellular processes. To assess whether the bacterium *W. chitiniclastica* contributes to delayed wound healing without preempting cellular mechanisms, a wound healing assay was performed as previously described to monitor epithelial gap closure (Liang et al., 2007; Pinto et al., 2020). This assay has been widely used in wound closure studies (Akram et al., 2013; Parvathaneni et al., 2020). A significantly reduced growth and migration rate was observed for all cells infected with *W. chitiniclastica* strains compared to normal growth and wound closure in non-infected controls. These findings are very important and strikingly important. Previous studies have shown that microbiome variation in diabetic foot wounds and pathogen diversity is associated with healing outcomes (Kalan et al., 2019). Moreover, pathogenic strains of *S. aureus* identified in non-healing wounds harbored antibiotic resistance genes and superantigens that amplify inflammatory responses and inhibit wound repair (Kalan et al., 2019). Interestingly, *Pseudomonas aeruginosa* can establish robust and persistent infections in diabetic wounds, irrespective of biofilm formation. A key factor contributing to tissue damage is the type III secretion system (T3SS), which negatively affects collagen synthesis and connective tissue regeneration, thereby significantly delaying wound healing in diabetic ulcers (Goldufsky et al., 2015). In addition to well-characterized pathogens, *W. chitiniclastica* may also influence cell migration and wound healing. One potential mechanism, analogous to findings by Goldufsky et al. (2015) involves the type II secretion system (T2SS), which may play a role in W. chitiniclastica strains (Goldufsky et al., 2015). T2SS are known to contribute to tissue destruction and are associated with cellular damage mediated by proteases and lipases (Sandkvist et al., 2001). Proteins secreted via T2SS are generally associated with tissue degradation and cell injury, including proteases, cellulases, pectinases, phospholipases, lipases, and various toxins (Sandkvist et al., 2001). According to Kopf et al. (2022) the T2SS in W. chitiniclastica is not primarily involved in adhesion (Durand et al., 2003) or toxin secretion but may instead mediate the secretion of chitinase, which could potentially contribute to delayed wound healing and should be investigated in future studies (Kopf et al., 2022).

However, several limitations of this study should be acknowledged. First, the findings are based on experiments using only four isolates of *W. chitiniclastica*. Therefore, the results should be considered preliminary and may not fully represent the intraspecies variability of this organism. Nevertheless, the observed consistent trends across the investigated isolates provide valuable initial insights and support further investigation of the underlying mechanisms. Additionally, this study was performed exclusively under nutrient-rich *in vitro* conditions. The behavior of *W. chitiniclastica* under nutrient-limited or environmental stress conditions was not assessed. Future studies using nutrient-restricted media could provide further insights into bacterial adaptation, survival strategies, and metabolic responses under unfavorable environmental conditions. Furthermore, validation at the protein level and *in vivo* models will be required to further elucidate the biological relevance and underlying mechanisms of the observed effects. Although the present study provides initial insights into the interaction of *W. chitiniclastica* with host systems under controlled *in vitro* conditions, additional approaches are necessary to determine whether these responses are representative of more complex physiological environments. Protein-level analyses, including the characterization of secreted bacterial factors or host-derived responses, could help to further clarify the molecular mechanisms involved and complement the findings obtained in this study. In addition, in vivo models will be essential to evaluate the relevance of these observations under clinically relevant conditions. Planned murine wound infection models, including studies in diabetic mice, may provide further insights into bacterial behavior, host-pathogen interactions, and the contribution of *W. chitiniclastica* to wound-associated infections. Such models will help to determine whether the effects observed *in vitro* reflect processes occurring in vivo and may provide a more comprehensive understanding of the pathogenic potential of this organism.

## 5 Conclusion

In conclusion, *W. chitiniclastica* appears to contribute to impaired wound closure and persistent inflammation in diabetic foot ulcers, as evidenced by delayed cell migration *in vitro*. This effect may be mediated by reduced epithelial cytokine expression of TSLP, IL-25 and IL-33, which can further delay the wound healing process. Additionally, the bacterium induces a pronounced proinflammatory response during the late stages of wound healing, with shifts in monocytic subsets from pro- to anti-inflammatory phenotypes being further exaggerated under glucose-driven conditions. Notably, significant impairment of wound closure was observed *in vitro* in cell line models, and the demonstrated tolerance of *W. chitiniclastica* to high-glucose environments suggests that glucose availability may modulate its pathogenic impact. Considering that subspecies formation often reflects environmental adaptation, potential adaptation of *W. chitiniclastica* to the human host warrants further investigation at both phenotypic and immunological levels. Future experimental studies using murine models of chronic diabetic wounds will be essential to fully elucidate these mechanisms.

## Data Availability

The datasets presented in this study can be found in online repositories. The names of the repository/repositories and accession number(s) can be found in the article/supplementary material.

## Footnotes

1 DSM 105838. Available from: https://bacdive.dsmz.de/pdf-view/132879?doi=10.13145%2Fbacdive132879.20260601.11.

## Abbreviations

A549: human alveolar epithelial cell line
ATCC: American type culture collection
AUC: area under the curve
CCL2: C-C motif chemokine ligand 2 (monocyte chemoattractant protein-1)
CCL7: C-C motif chemokine ligand 7 (monocyte chemoattractant protein-3)
CFU: colony-forming units
CO₂: carbon dioxide
CT: threshold cycle
CX3CR1: C-X3-C motif chemokine receptor 1
ddH₂O: double-distilled water
DFU: diabetic foot ulcer
DSMZ: German collection of microorganisms and cell cultures (Deutsche Sammlung von Mikroorganismen und Zellkulturen)
*E. coli*: *Escherichia coli*
FCS: FETAL calf serum
GAPDH: glyceraldehyde-3-phosphate dehydrogenase
HEPES: 4-(2-hydroxyethyl)-1-piperazineethanesulfonic acid
IL: interleukin
LB broth: Luria–Bertani broth
LSU: light scattering unit
Ly6G6C: lymphocyte antigen 6 family member G6C
MALDI-TOF MS: matrix-assisted laser desorption/ionization time-of-flight mass spectrometry
McF: McFarland standard
MOI: multiplicity of infection
MTSS: medial tibial stress syndrome
NC: negative control
OD600: optical density measured at 600 nm
PAMPs: pathogen-associated molecular patterns
PBS: phosphate-buffered saline
PC: positive control
P/S: penicillin/streptomycin
qPCR: quantitative real-time polymerase chain reaction
RNA: ribonucleic acid
rpm: revolutions per minute
rRNA: ribosomal ribonucleic acid
RT-PCR: reverse transcription polymerase chain reaction
SEM: standard error of the mean
*S. aureus*: *Staphylococcus aureus*
T2SS: type II secretion system
THP-1: human monocytic leukemia cell line
TSLP: thymic stromal lymphopoietin
T3SS: type III secretion system.
