# Correction: *Wohlfahrtiimonas chitiniclastica*: a potential disruptor of wound healing and glucose metabolism in diabetic foot ulcers

**DOI:** 10.3389/fmicb.2026.1941007

**Published:** 2026-08-20

**Authors:** Theresa Kurze, Muhammed Afthab Tharakathel Abdul Majeed, Percy Schröttner, Hani Harb

**Affiliations:** 1 Institute for Medical Microbiology and Virology, Faculty of Medicine and University Hospital Carl Gustav Carus, Technische Universität Dresden, Dresden, Germany; 2 Institute for Clinical Chemistry and Laboratory Medicine, Faculty of Medicine and University Hospital Carl Gustav Carus, Technische Universität Dresden, Dresden, Germany

**Keywords:** diabetes, diabetic ulcers, immune reaction, *Wohlfahrtiimonas chitiniclastica*, wound healing

The figures and captions are incorrectly placed and ordered, with several captions not matching their respective figures.

There was a mistake in the caption of Figures 1, 2, 3 and 4 as published. All figures and captions are in the wrong order. The right place of the figures belonging to the numbers is stated below. The corrected captions of Figures 1–4 appear below.

**Figure 1:**
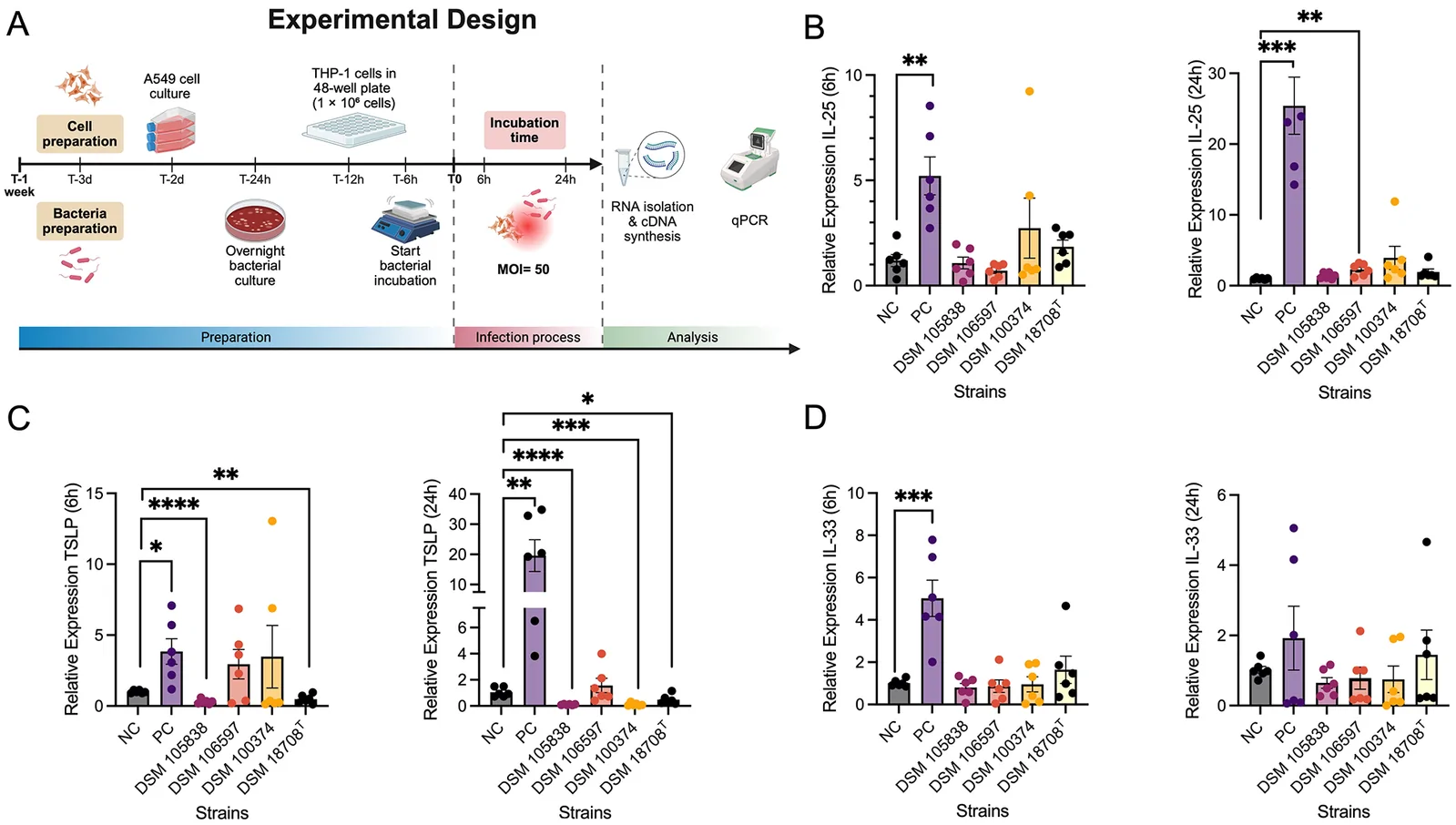
*W. chitiniclastica* induces alarmin gene expression in A549 cells. Experimental design **(A)**. Relative mRNA expression of TSLP **(B)**, IL-25 **(C)**, and IL-33 **(D)** in A549 cells infected with *W. chitiniclastica* (MOI 50) for 6 h and 24 h. Untreated cells served as negative control (NC); *E. coli–*infected cells as positive control (PC). Gene expression was analyzed by qPCR using the ΔΔCT method with GAPDH as reference. Data are mean ± SEM (*n* = 6 Biological replicates). Statistical tests: one-way ANOVA with Tukey's *post-hoc* analysis. Data representative of two independent experiments. ^*^p < 0.05, ^**^p < 0.01, ^***^p < 0.001, ^****^p < 0.0001.

**Figure 2:**
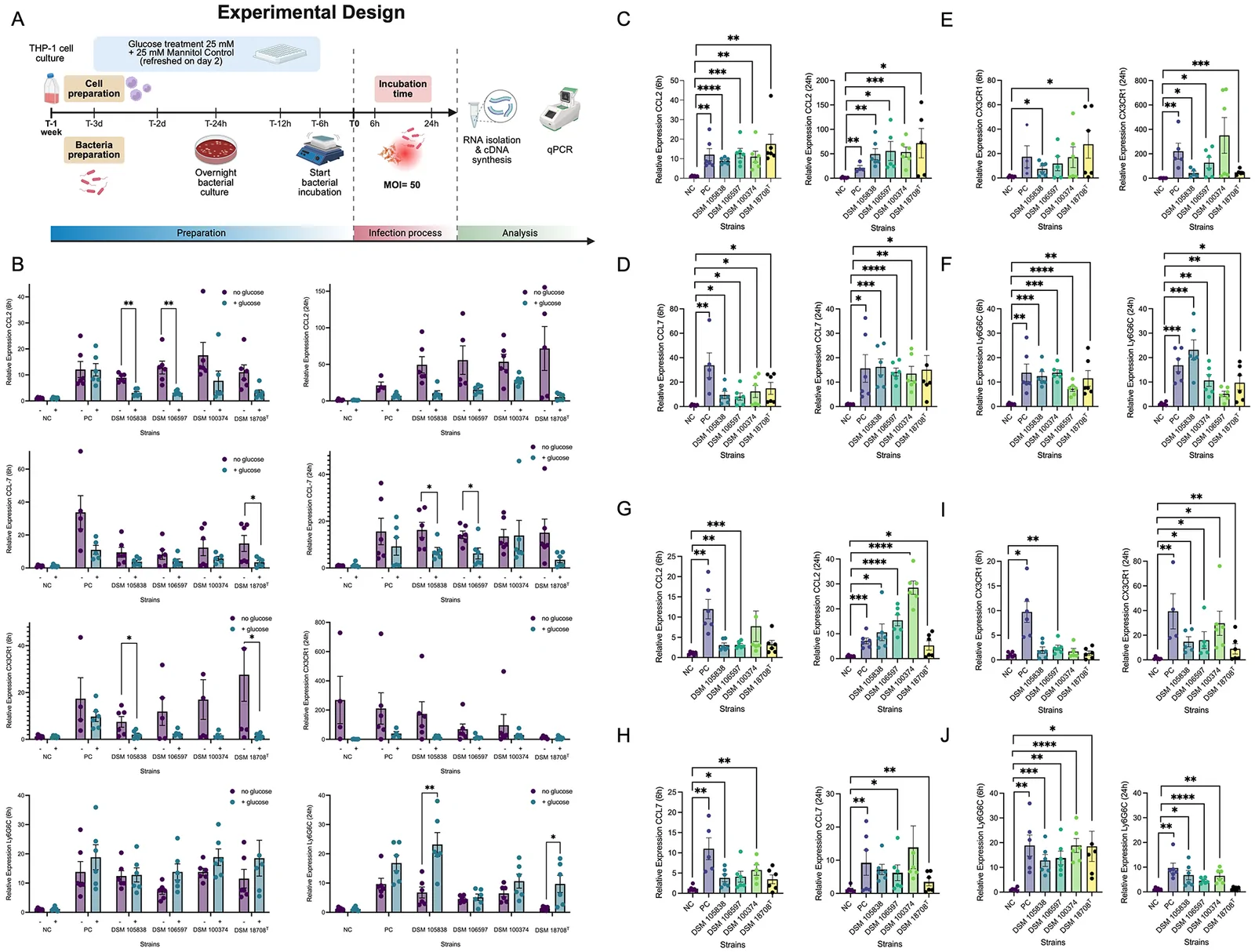
*W. chitiniclastica* induces chemokine and immune marker expression in THP-1 cells under glucose conditions. Experimental design **(A)**. Relative mRNA expression of CCL2 **(C)**, CCL7 **(D)**, CX3CR1 **(E)**, and LY6G6C **(F)** in THP-1 cells infected with W. chitiniclastica (MOI 50) for 6 h and 24 h following 3-day pretreatment with 25 mM glucose or 25 mM mannitol (osmotic control): Direct comparisons between glucose-treated and untreated cells are illustrated **(B)**. Detailed gene expression data for individual genes, shown exclusively for glucose-treated conditions and separated by 6 and 24 h, are provided **(G–J)**. Untreated cells served as negative control (NC); *Escherichia coli*–infected cells as positive control (PC). Gene expression was analyzed by qPCR using the ΔΔCT method with GAPDH as reference. Data are mean ± SEM (*n* = 6 biological Replicates). Statistical tests: one-way ANOVA with Tukey's *post-hoc* analysis. Data representative of two independent experiments. ^*^p < 0.05, ^**^p < 0.01, ^***^p < 0.001, ^****^p < 0.0001.

**Figure 3:**
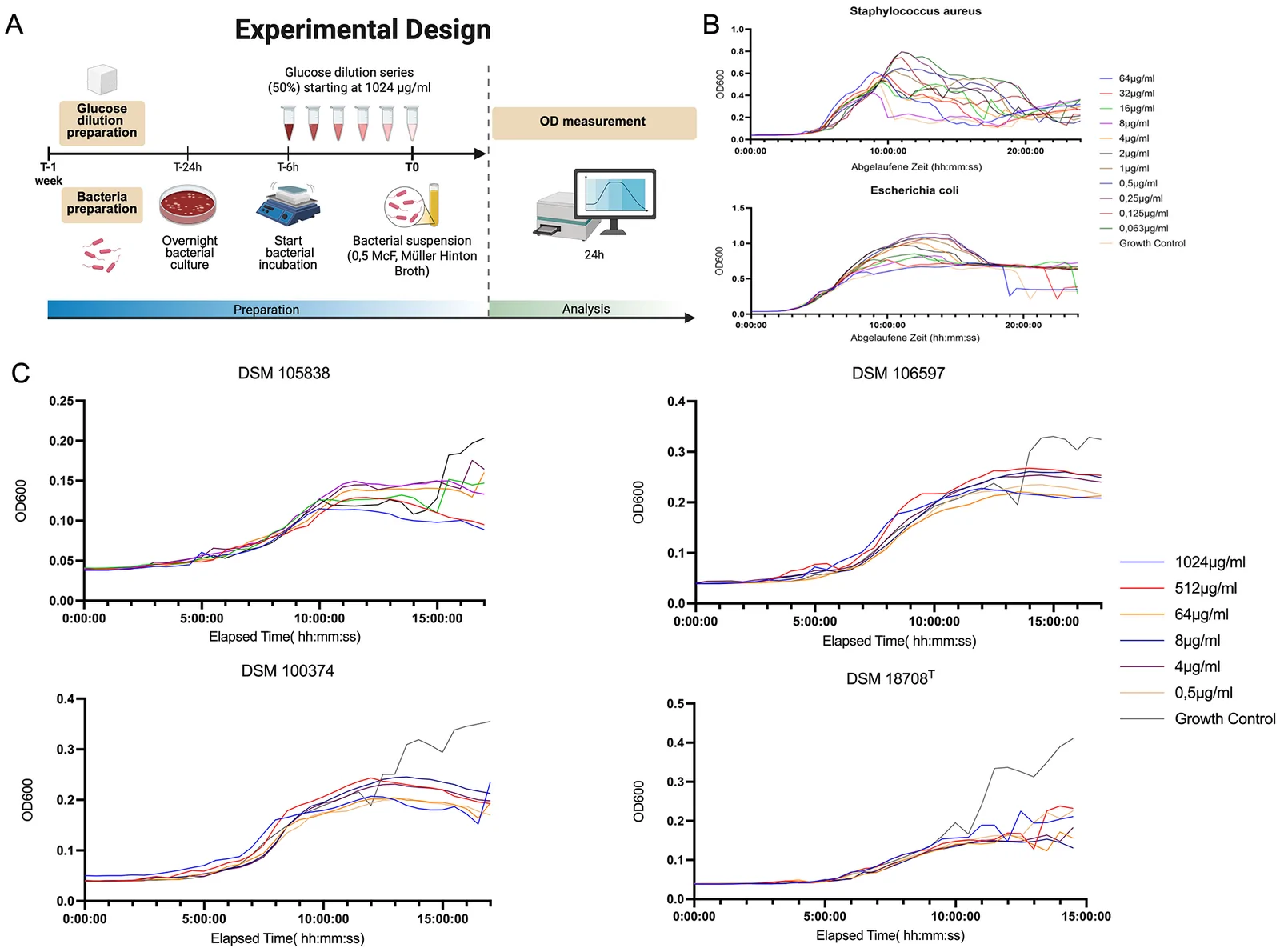
Growth kinetics of *W. chitiniclastica* compared to glucose-utilizing bacteria. Experimental design **(A)**. Growth curves of glucose-utilizing strains *Staphylococcus aureus* and *Escherichia coli* **(B)**. Growth curves of *W. chitiniclastica* strains **(C)**.

**Figure 4:**
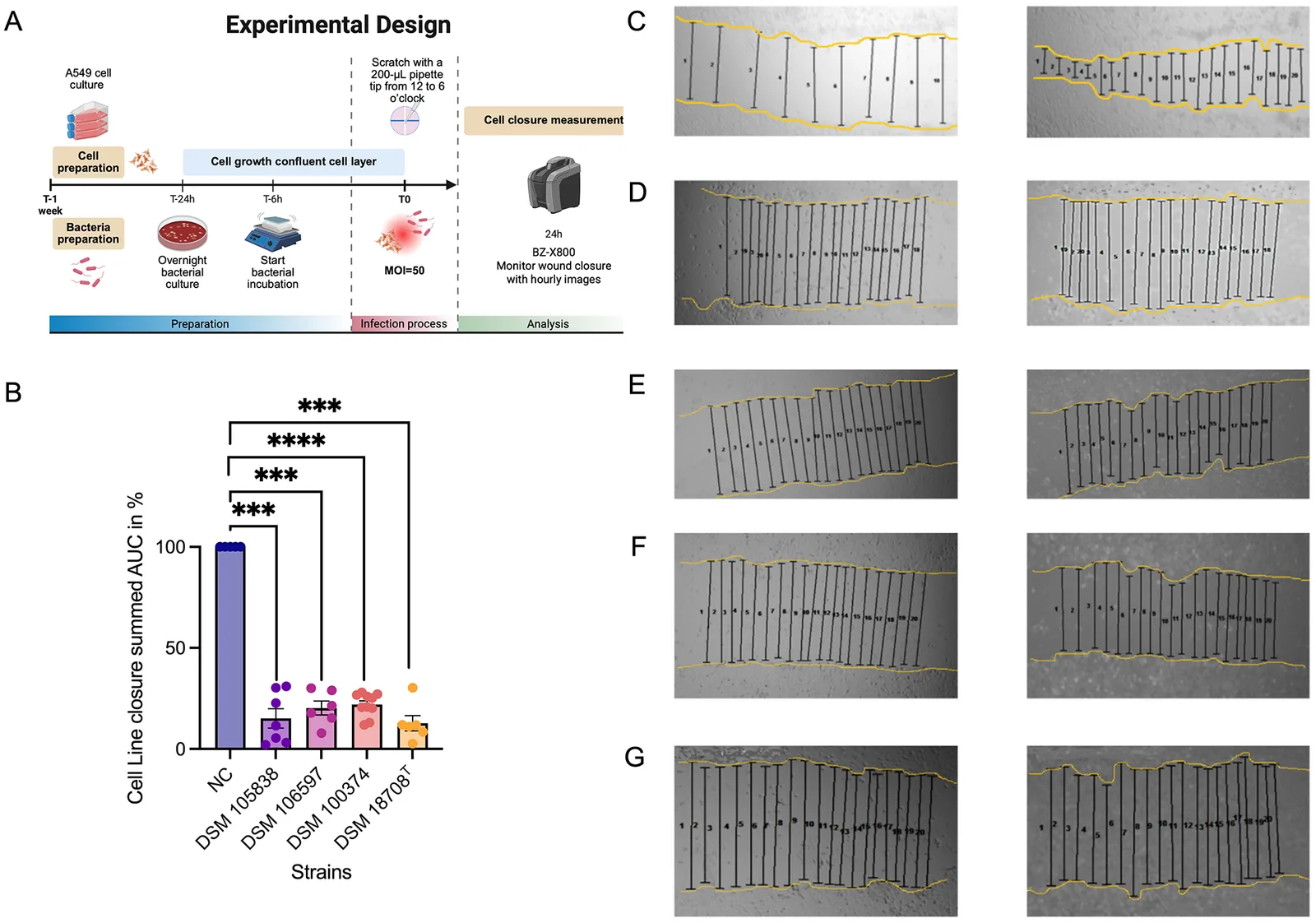
*W. chitiniclastica* modulates epithelial cell migration. Experimental design of the wound healing assay in A549 cells **(A)**. Quantification of wound closure expressed as percentage closure over time and cumulative area under the curve (AUC) **(B)**. Representative images of wound closure at 0 h and 24 h in untreated control cells **(C)** and cells exposed to *W. chitiniclastica* strains DSM 105838 **(D)**, DSM 106597 **(E)**, DSM 100374 **(F)**, and DSM 18708T **(G)**. (*n* = 5 biological Replicates), Statistical tests: one-way ANOVA with Tukey's *post-hoc* analysis. Data representative of two independent experiments. ^*^p < 0.05, ^**^p < 0.01, ^***^p < 0.001, ^****^p < 0.0001.

The figures and figure captions were in the wrong order in the PDF and online version of this paper.

The figure noted as Figure 1 is supposed to be Figure 4. The figure noted as Figure 2 has to be Figure 1. The figure noted as Figure 3 is correctly Figure 3. The figure noted as Figure 4 is supposed to be Figure 2. All figures are supposed to be in the results section.

Figure 1 belongs to the end of 3.1. Figure 2 belongs in the end of Sections 3.3. Figure 3 Belongs in the end of Section 3.4. Figure 4 belongs in the end of Section 3.5. The order has now been corrected.

The original version of this article has been updated.

